# ISOC1 Modulates Inflammatory Responses in Macrophages through the AKT1/PEX11B/Peroxisome Pathway

**DOI:** 10.3390/molecules27185896

**Published:** 2022-09-11

**Authors:** Xiaoyuan Lin, Qingting Zhao, Beibei Fu, Yan Xiong, Shanfu Zhang, Shiyao Xu, Haibo Wu

**Affiliations:** School of Life Sciences, Chongqing University, Chongqing 401331, China

**Keywords:** ISOC1, PEX11B, AKT1, peroxisome, inflammation

## Abstract

Inflammation underlies a variety of physiological and pathological processes and plays an essential role in shaping the ensuing adaptive immune responses and in the control of pathogens. However, its physiological functions are not completely clear. Using a LPS-treated RAW264.7 macrophage inflammation model, we found that the production of inflammatory cytokines in ISOC1-deficient cells was significantly higher than that in the control group. It was further proved that ISOC1 deficiency could activate AKT1, and the overactivation of AKT1 could reduce the stability of PEX11B through protein modification, thereby reducing the peroxisome biogenesis and thus affecting inflammation. In this study, we reported for the first time the role of ISOC1 in innate immunity and elucidated the mechanism by which ISOC1 regulates inflammation through AKT1/PEX11B/peroxisome. Our results defined a new role of ISOC1 in the regulatory mechanism underlying the LPS-induced inflammatory response.

## 1. Background

Inflammation is closely related to the occurrence of many diseases, such as obesity, type 2 diabetes, and cancer [1,2,3]. The macrophage-mediated inflammatory response is part of the innate immune system, which protects the host against invading pathogens [4]. Lipopolysaccharide (LPS) is the main component of the cell wall of Gram-negative bacteria, which stimulates the inflammatory response through macrophages. In this paper, macrophages stimulated by LPS were used as a model of bacterial infection. During the inflammatory response, Toll-like receptor 4 recognizes extracellular LPS and activates inflammatory signaling pathways, leading to the production of inflammatory mediators [5,6,7]. However, inflammation-related regulatory pathways are both important and complex, and there are still new regulatory mechanisms worth exploring, to provide new ideas for the treatment of inflammation-related diseases.

Isochorismatase domain-containing 1 (ISOC1), a member of the Isochorismatase hydrolase family, is a new peroxisomal constituent in human liver peroxisomes [8,9,10]. Some studies have shown that ISOC1 plays an important role in the regulation of apoptosis and the migration of various cancer cells [11,12,13]. A recent study showed that the downstream signaling pathways mediated by ISOC1 were mainly related to inflammation through RNA sequencing analysis [14]. However, the role of ISOC1 in inflammation is still unknown.

As a member of AKT family, AKT1 Serine/Threonine Kinase 1(AKT1) has been reported to be a critical signaling node in the context of inflammation in multiple studies. For instance, AKT1 regulates glycolysis and the function of myeloid-derived suppressor cells through HIF1α in inflammation and cancer [15]. lncRNA MALAT1 can promote the extent of AKT1 phosphorylation and increase skeletal muscle cell apoptosis and inflammatory responses [16]. As an essential metabolic organelle, peroxisome plays an important role in the synthesis and turnover of complex lipids and reactive species, and peroxisomes have recently been identified as pivotal regulators of inflammation during infection [17]. There are 31 known peroxins involved in various aspects of peroxisome biogenesis, including peroxisome generation, division, and matrix and membrane protein import [18]. In this study, we found that ISOC1 regulated the inflammatory response by affecting AKT1 phosphorylation and peroxisomes upon LPS treatment.

The significance of this study is to reveal the role of ISOC1 in the regulation of inflammatory response. ISOC1 regulates peroxisome biogenesis through the AKT1/Peroxisomal Biogenesis Factor 11 Beta (PEX11B)/peroxisome pathway to regulate inflammatory response. The phosphorylation of AKT1 resulted in the degradation of PEX11B by affecting the protein modification of PEX11B. We established an ISOC1/AKT1/PEX11B/peroxisome axis to regulate inflammation, which provides new ideas for the treatment of inflammation-related diseases.

## 2. Results

### 2.1. ISOC1 Expression Is Increased under LPS Stimulation and ISOC1 Regulates the Expression of Inflammatory Cytokines

**Figure 1:**
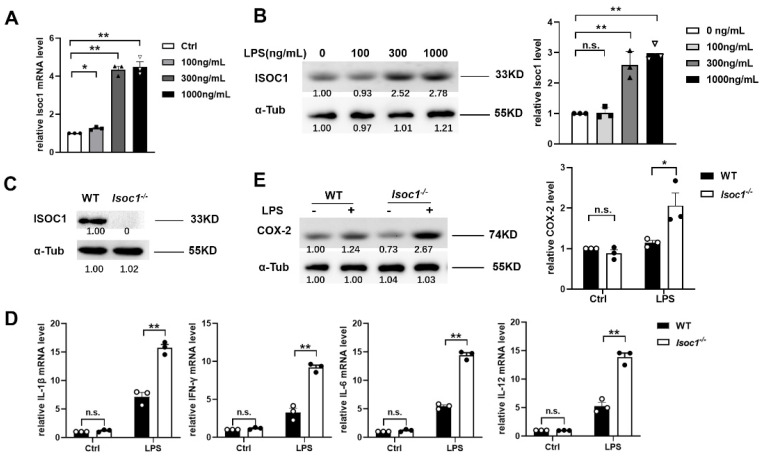
LPS induces the expression of ISOC1, and ISOC1-deficient macrophages show a higher inflammation with LPS treatment. (**A**,**B**) RAW264.7 cells were treated with specified concentration of LPS; ISOC1 expression levels were measured by qRT-PCR (**A**) and Western blotting (**B**). (**C**) The protein levels of ISOC1 were measured by WB in WT and ISOC1-deficient cell lines. (**D**) qRT-PCR was used to detect the expression levels of IL-1β, IFN-γ, IL-6, and IL-12 with 300 ng/mL LPS for 24 h. (**E**) Western blotting was used to detect COX-2 protein levels in wild-type and ISOC1-deficient RAW264.7 stimulated with 300 ng/mL LPS for 24 h. (**A**,**D**) were representative of one experiment with at least three independent biological replicates; a single data point represented one technical repeat. All data are shown as mean ± s.e.m. (*n* = 3). * *p* < 0.05, ** *p* < 0.01; n.s. not significant. ANOVA followed by Bonferroni post hoc test was used for data analysis.

To investigate the effect of LPS on ISOC1, we firstly performed qRT-PCR analysis and Western blotting (WB) to measure the expression level of ISOC1 in wild-type (WT) RAW264.7 macrophages. The results showed that ISOC1 expression increased along with the consistence increase in LPS concentration (Figure 1A,B). In order to study the role of ISOC1 in inflammatory response, we established an ISOC1 knockout (Isoc1^-/-^) RAW264.7 cell line with CRISPR/Cas9 (Figure 1C) and overexpressed ISOC1 using a pCMV-Isoc1 construct. Then, we examined the levels of several proinflammatory/anti-inflammatory factors and the expression of COX-2, an inflammatory mediator protein 1 in different groups. It was found that after LPS treatment, the COX-2 level and the expression of proinflammatory factors, including IL-1β, IFN-γ, IL-6, and IL-12, increased significantly in Isoc1^-/-^ compared to the WT groups (Figure 1D,E), while anti-inflammatory factors, including IL-4 and IL-10, decreased significantly in Isoc1^-/-^ compared to the WT groups (Figure S1A). We also tested cell death in Isoc1^-/-^ cells, and the results revealed that after LPS treatment, the Isoc1^-/-^ cells showed a higher apoptosis rate compared with the WT cells (Figure S1B). Taken together, Isoc1^-/-^ showed a higher inflammatory response and apoptosis than WT after LPS treatment.

### 2.2. ISOC1 Mediates Macrophage Inflammatory Response via AKT1 and NF-κB Pathway

**Figure 2:**
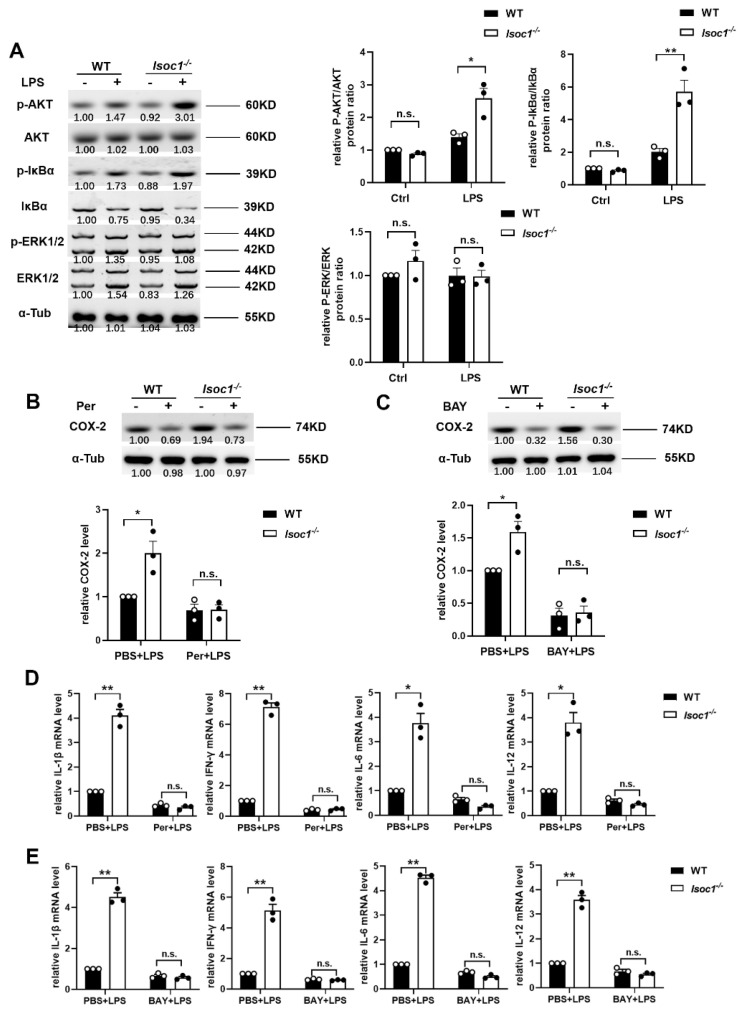
ISOC1 mediates macrophage inflammatory response via AKT1 and NF-κB pathway. (**A**) Wild-type or ISOC1-deficient RAW264.7 cells were stimulated with 300 ng/mL LPS for 24 h. Cell lysates were collected, and western blotting was used to detect the phosphorylation levels of AKT1, IκBα, and ERK. (**B**,**C**) ISOC1-deficient RAW264.7 cells were pretreated with 10 μM Perifosine for 12 h (**B**) or 10 μM BAY 11-7082 for 1.5 h (**C**) and stimulated with 300 ng/mL LPS for 24 h. Expressions of the COX-2 protein were detected by Western blotting. (**D**,**E**) Wild-type and ISOC1-deficient RAW264.7 cells were pretreated with 10 μM Perifosine for 12 h (**D**) or 10 μM BAY 11-7082 for 1.5 h (**E**) and stimulated with 300 ng/mL LPS for 24 h. qRT-PCR was used to detect the expression levels of IL-1β, IFN-γ, IL-6, and IL-12. All data are shown as mean ± s.e.m (*n* = 3). * *p* < 0.05, ** *p* < 0.01; n.s. not significant. Two-way ANOVA followed by Bonferroni post hoc test was used for data analysis.

It has been reported that AKT1 and NF-κB are closely associated with inflammatory response in previous studies [19,20]. In order to explore the pathways by which ISOC1 regulates the expression of inflammatory factors, the phosphorylation levels of AKT1, IκBα, and ERK were tested in WT and Isoc1^-/-^ with PBS or LPS treatment. The results showed that after LPS stimulation, the phosphorylation levels of AKT1 and IκBα were more significantly upregulated in ISOC1-deficient RAW264.7 cells (Figure 2A). To determine whether AKT1 and NF-κB are responsible for the increased expression of inflammatory cytokines and COX-2 in Isoc1^-/-^, the cells were pretreated with Per (Perifosine, AKT1 inhibitor) or BAY (BAY 11-7082, NF-κB inhibitor) and stimulated with LPS. We found that in Per- or BAY- treated groups, the COX-2 level showed no significant difference between Isoc1^-/-^ and WT; however, in PBS-treated groups, their levels were higher in Isoc1^-/-^ than in WT groups (Figure 2B,C). Then, the expression of inflammatory factors was detected by qRT-PCR. Additionally, the results showed that in PBS-treated groups, their levels were higher in Isoc1^-/-^ than in WT groups; however, in Per- or BAY- treated groups, their levels showed no significant difference between Isoc1^-/-^ and WT (Figure 2D,E). Since it has been reported that ISOC1 knock-down suppresses cell proliferation in pancreatic cancer and colon cancer cells [11,12], we studied the effect of ISOC1 deletion on cell proliferation in RAW 264.7 cells treated with LPS. The results showed that ISOC1 deletion did not exert a significant influence on cell proliferation in our model (Figure S1C), indicating that the function of ISOC1 on inflammatory response is independent of cell proliferation. Together, these data indicate that the function of ISOC1 deletion in the regulation of inflammation response depends on AKT1 and the NF-κB pathway in LPS-stimulated macrophages. Interestingly, ISOC1 overexpression also promoted the secretion of inflammatory factors (Figure S1D,E). Using WB, the P-AKT1 level was tested in ISOC1-overexpressing cells. The results in Figure S1D showed that after overexpressing ISOC1 using a vector, the P-AKT1 level was reduced. Since P-AKT1 reduction has also been reported to be related to activation of inflammatory response [21], we speculate that there might be another pathway through which ISOC1 overexpression inhibits P-AKT1 and leads to inflammatory response activation. However, more experiments will be needed to study the mechanism in detail.

### 2.3. The Response to LPS Stimulation Reduces Peroxisomal Production in Isoc1^-/-^ Macrophages

**Figure 3:**
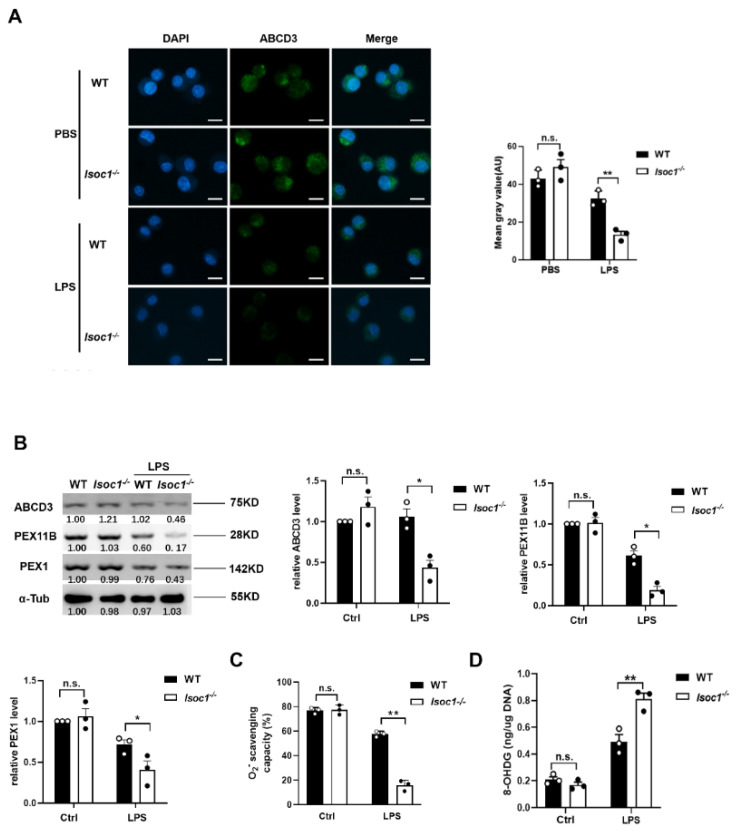
After LPS treatment, peroxisome level decreases more significantly in ISOC1-deficient cells than in wild-type cells. Wild-type or ISOC1-deficient RAW264.7 cells were treated with LPS (300 ng/mL) for 24 h. (**A**) Peroxisomes were labeled with ABCD3 and were observed with fluorescence microscopy. Scale bar = 10 μm. Mean gray values were analyzed by Image J v1.51 (Wayne Rasband, Bethesda, MD, USA). (**B**) Cell lysates were subjected to WB with antibodies to ABCD3, PEX11B, and PEX1. (**C**) O2—scavenging capacity was measured using a superoxide anion scavenging capacity assay Kit. (**D**) The content of 8-OHDG was analyzed by an oxidative RNA Damage ELISA Kit. All data are shown as mean ± s.e.m (*n* = 3). * *p* < 0.05, ** *p* < 0.01; n.s. not significant. Two-way ANOVA followed by Bonferroni post hoc test was used for data analysis.

As essential metabolic organelles, peroxisomes have recently been identified as a pivotal regulators of inflammation during infection [17]; we thus tested the peroxisome levels in WT and Isoc1^-/-^ treated with LPS. As a peroxisomal membrane protein, ABCD3 has been widely used as the marker protein of peroxisomes in previous studies [22,23,24,25,26] and was used to label peroxisomes in this study. The results showed that, in LPS-treated groups, the fluorescence intensity of peroxisomes of Isoc1^-/-^ was significantly lower than that of the WT (Figure 3A). Then, we examined the peroxisomal associated proteins, including ABCD3, PEX11B, and PEX1. The results showed that after LPS stimulation, the expression of these proteins in Isoc1^-/-^ was lower than that of the WT cells (Figure 3B), and this influence was eliminated after overexpressing ISOC1 in Isoc1^-/-^ cells (Figure S1F). We also knocked down Pex11b using shRNA in RAW264.7 cells and tested its influence on peroxisomes and inflammatory response to LPS. The knock down efficiency was evaluated using WB (Figure S2A), and the immunofluorescence staining results showed that Pex11b knock-down led to a decrease in peroxisome fluorescence intensity (Figure S2B), which is consistent with previous reports [27,28,29]. The qPCR results proved that after LPS stimulation, Pex11b knock-down enhanced inflammatory response (Figure S2C). Because peroxisomes play an important role in oxidative stress [30], we detected the generation of O2− and formation of 8-OHDG, a ubiquitous marker of oxidative stress, under the stimulation of LPS in Isoc1^-/-^ and WT. We found that after LPS stimulation, the generation of O2− and the formation of 8-hydroxyguanosine increased significantly in Isoc1^-/-^ macrophages compared to that in WT (Figure 3C,D). Taken together, these results indicate that under LPS stimulation, ISOC1 deletion reduces peroxisome levels in macrophages and enhances the level of oxidative stress.

### 2.4. AKT1 Regulates Peroxisome Biogenesis

**Figure 4:**
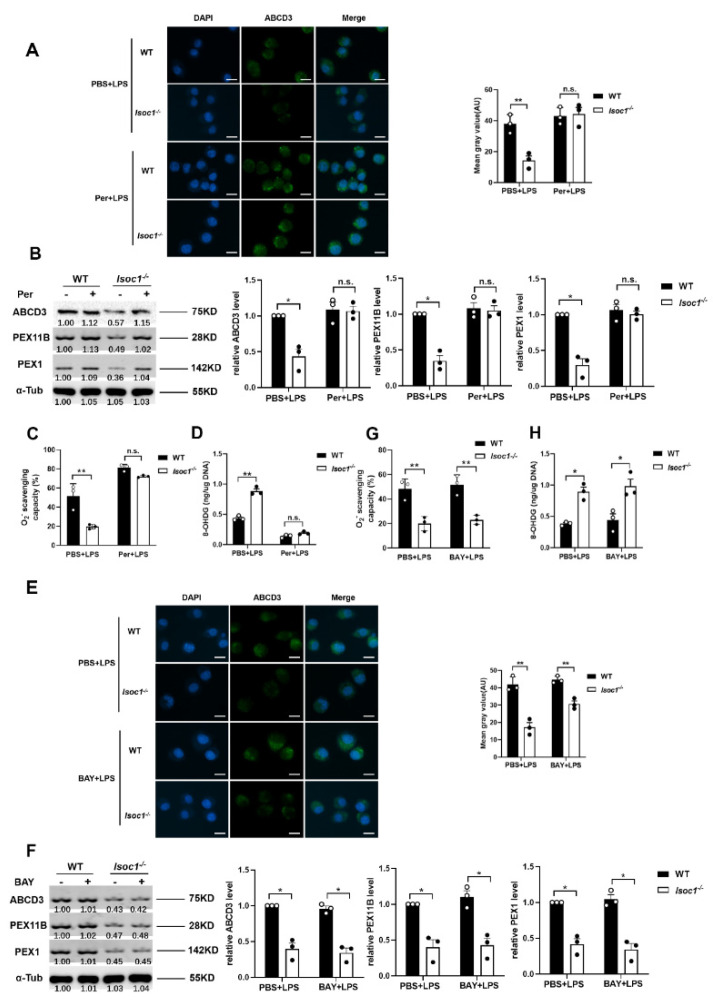
ISOC1 affects peroxisomes through the AKT1 pathway but not the NF-κB pathway. Wild-type or ISOC1-deficient RAW264.7 were pretreated with 10 μM Perifosine for 12 h or 10 μM BAY 11-7082 for 1.5 h, stimulated with 300 ng/mL LPS for 24 h. (**A**,**E**) Peroxisomes were labeled with ABCD3 and were observed with fluorescence microscopy. Scale bar = 10 μm. Mean gray value were analyzed by Image J. (**B**,**F**) WB was used to detect the expression levels of ABCD3, PEX11B, and PEX1. (**C**,**G**) O2− scavenging capacity was measured using a superoxide anion scavenging capacity assay Kit. (**D**,**H**) The content of 8-OHDG was analyzed by an oxidative RNA Damage ELISA Kit. All data are shown as mean ± s.e.m (*n* = 3). * *p* < 0.05, ** *p* < 0.01, n.s. not significant. Two-way ANOVA followed by Bonferroni post hoc test was used for data analysis.

In order to investigate whether the effect of ISOC1 on peroxisomes is mediated by AKT1 and NF-κB pathways, the following experiments were designed. Per, which can specifically bind to the pleckstrin homology domain of AKT1 to prevent its membrane translocation [31,32,33], was used as an AKT1 inhibitor in this study. The results showed that under LPS stimulation, ISOC1 deficiency reduced peroxisome levels. After pretreatment with Akt1 inhibitors, the levels of peroxisomes were restored in Isoc1^-/-^ cells (Figure 4A,B). In PBS groups, the levels of O2− and 8-OHDG were significantly increased in ISOC1-deficient cells than in WT groups, while after treatment with the AKT1 inhibitors, the two groups showed no significant difference (Figure 4C,D). On the other hand, the peroxisome level was not restored in Isoc1^-/-^ cells pretreated with NF-κB inhibitors (Figure 4E,F). Similarly, in PBS-treated groups, the levels of O2− and 8-OHDG were significantly increased in Isoc1^-/-^ cells, and after pretreatment with the NF-κB inhibitors, their levels showed a similar trend (Figure 4G). Taken together, these results suggest that the regulation of ISOC1 on peroxisomes depends on AKT1 activation.

### 2.5. AKT1 Promotes the Stability of PEX11B through Phosphorylation and Ubiquitination

**Figure 5:**
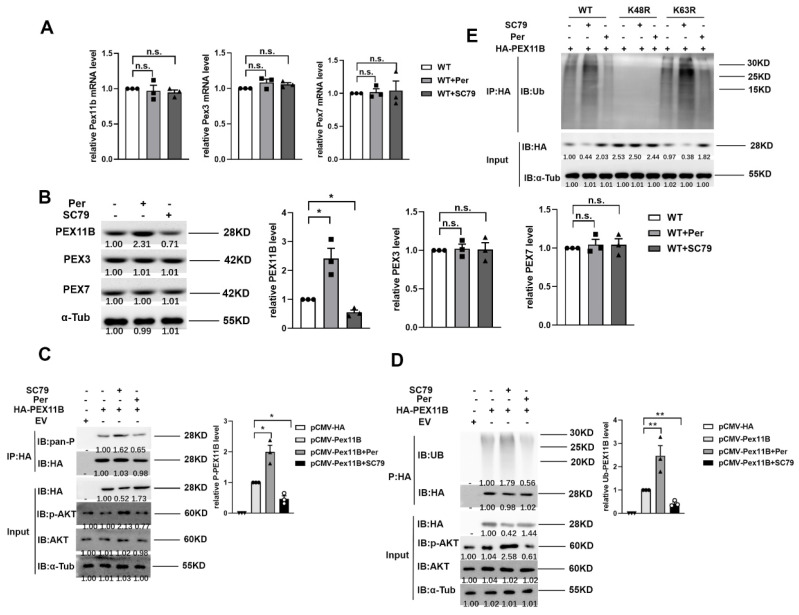
AKT1 phosphorylation regulates the modification and stability of PEX11B. (**A**) Wild-type RAW264.7 was pretreated with 10 μM Perifosine for 12 h or 10 μM SC79 for 2 h, stimulated with 300 ng/mL LPS for 24 h. qRT-PCR was used to detect the mRNA levels of PEX11B, PEX3, and PEX7. (**B**) Wild-type RAW264.7 was pretreated with 10 μM Perifosine for 12 h or 10 μM SC79 for 2 h, stimulated with 300 ng/mL LPS for 24 h. Western blotting was used to detect the protein levels of PEX11B, PEX3, and PEX7. (**C**,**D**) HA-labeled PEX11B or pCMV-HA was transfected into cells for 24 h, pretreated with 10 μM Perifosine for 12 h or 10 μM SC79 for 2 h, and stimulated with LPS for 24 h. The phosphorylation level (**C**) and ubiquitin level (**D**) of PEX11B were detected by Co-IP. (**E**) Cells were transfected with different ubiquitin mutants and HA-labeled PEX11B, pretreated with 10 μM Perifosine for 12 h or 10 μM SC79 for 2 h, and stimulated with LPS for 24 h. The interaction between PEX11B and ubiquitin was detected by Co-IP. All data are shown as mean ± s.e.m (*n* = 3). * *p* < 0.05, ** *p* < 0.01, n.s. not significant. One-way ANOVA followed by Bonferroni post hoc test was used for data analysis.

**Figure 6:**
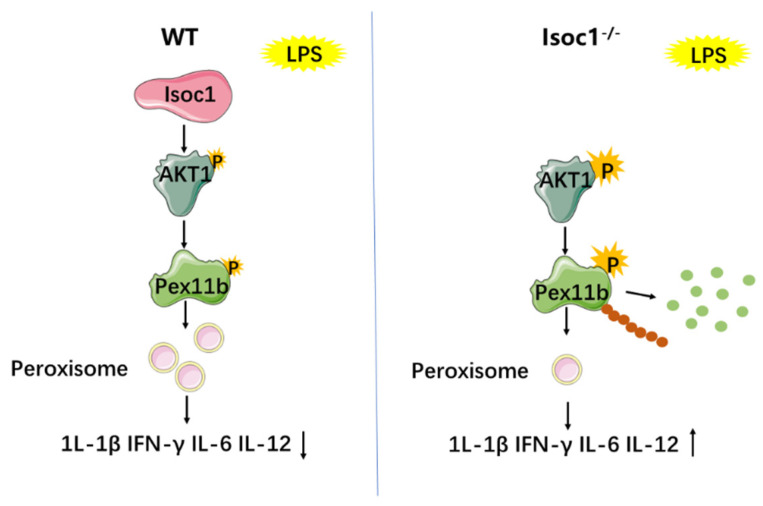
ISOC1 affects the expression of inflammatory cytokines through the AKT1 /PEX11B/peroxisome pathway. Compared with the wild-type macrophages, ISOC1-deficient macrophages exhibited a hyperphosphorylation of AKT1 under the stimulation of LPS. The hyperphosphorylation of AKT1 enhanced the phosphorylation and ubiquitination of PEX11B, weakening the stability of PEX11B protein. As an important protein that regulates the growth and division of peroxisomes, PEX11B degradation leads to a decrease in peroxisomes and an increase in the production of IL-1β, IFN-γ, IL-6, and IL-12.

We further studied the effect of AKT1 on peroxisomes. Among the 16 known peroxins involved in peroxisome biogenesis in mammalians, we selected PEX3, PEX7, and PEX11B [18] and studied the influence of AKT1 activation on them. Per/SC79 is widely used in previous reports as an inhibitor/activator of AKT1 and was thus used in this study. Interestingly, SC79 can also bind to the pleckstrin homology domain of AKT1; however, SC79-bound AKT1 adopts a conformation favorable for phosphorylation by upstream protein kinases and activates AKT1 in the cytosol [34]. The result showed that after LPS stimulation, the mRNA levels of PEX3, PEX7, and PEX11B in cells pretreated with Per (AKT1 inhibitor) or SC79 (AKT1 activator) did no change significantly (Figure 5A). Interestingly, pretreatment with the AKT1 inhibitor in RAW264.7 cells significantly increased the protein level of PEX11B, while pretreatment with the activator did the opposite. The activators and inhibitors of AKT1 had no significant effect on the protein levels of PEX3 and PEX7. (Figure 5B). These results suggest that AKT1 activation may affect peroxisomes by influencing the degradation of PEX11B protein. Next, pCMV-HA or HA-tagged PEX11B was transfected into cells, and coimmunoprecipitation showed that the phosphorylation of PEX11B (Figure 5C) and the ubiquitination of PEX11B (Figure 5D) were enhanced by AKT1 activation and suppressed by AKT1 inhibition. We also examined PEX11b phosphorylation/degradation in ISOC1 knockout cells. As suspected, both the phosphorylation and ubiquitination level of Pex11b increased in Isoc1 knockout cells (Figure S2D). There are many known ubiquitination modifications, among which the K48-linked chain and K63-linked chain modifications are the most studied. Therefore, we used ubiquitin mutants K48R and K63R to study the way of ubiquitin chain linkage of PEX11B. The results showed that AKT1 promotes PEX11B degradation through ubiquitination at K48, instead of K63 (Figure 5E). These findings indicate that the activation of AKT1 regulated the posttranslational modification of PEX11B and reduced the content of PEX11B by promoting phosphorylation and ubiquitination, thereby regulating peroxisomes (Figure 6).

## 3. Methods

### 3.1. Cell Culture and Transfection

RAW264.7 cells were purchased from American Type Culture Collection (ATCC, TIB-71). The culture medium was composed of Dulbecco’s Modified Eagle’s Medium (DMEM, Gibco, San Jose, CA, USA) and 10% fetal bovine serum (Gibco). All cells were incubated at 37 °C and 5% CO_2_. Plasmid DNA was transfected into indicated cells using Lipofectamine 3000 Transfection Reagent (Invitrogen, Life Technologies, Carlsbad, CA, USA).

The ISOC1-deficient RAW264.7 cells was constructed using CRISPR/Cas9. Specifically, the open reading frame (ORF) of ISOC1 was interrupted to disrupt the translation of mRNAs using the CRISPR/Cas9 system. The target site for ISOC1 is 5′-CGACATGCACCGCAAATTCG-3′. In order to study the role of Isoc1 in inflammatory response, we constructed an inflammatory model by treating cells with 300 ng/mL LPS (Invitrogen, 00-4976-93) for 24 h. 

### 3.2. Plasmids Construction 

The encoding sequence of ISOC1 (NCBI accession number: NM_025478.3) was constructed into the pCMV-C-Flag vector (Beyotime, Shanghai, China). The forward primer sequence was 5′-AATTGAATTCATGGCGGCGGCGGAGC-3′, and the reverse primer sequence was 5′-TCGGGTCGACCACTTTAGAAAGCAGA-3′. The full-length coding sequence of PEX11B (NCBI accession number: NM_011069.3) was inserted into pCMV-C-HA vector (Beyotime). The forward primer was 5′-TACAGGATCCATGGACGCCTGGGTC-3′, and the reverse primer was 5′-TCATGAATTCGGGCTTGAGTCG-3′. The K48R and K63R mutants were kindly provided by Dr. Yonghui Zheng (Michigan State University). The wild-type Ubquitin was purchased from Addgene (Watertown, MA, USA). Pex11b shRNA was designed by BLOCK-iT RNAi Designer (https://rnaidesigner.thermofisher.com/, accessed on 10 June 2020). Both strands were annealed and ligated in pLKO.1 vector with T4 ligase. Sense strand: 5′-CCGGGCTGGATGTGCTCAGAAATGCTTCAAGAGAGCATTTCTGAGCACATCCAGCTTTTT-3′; antisense strand: 5′-AATTAAAAAGCTGGATGTGCTCAGAAATGCTCTCTTGAAGCATTTCTGAGCACATCCAGC-3′.

### 3.3. Real-Time Quantitative PCR

Total RNA was isolated from cells using TRIzol reagent, according to the manufacturer’s instructions; RNA was reverse transcribed using a PrimeScript RT Reagent Kit (Takara, Dalian, China). The expression of mRNAs was quantified using an SYBR Premix ExTaq II Kit (Takara). Real-time quantitative PCR (qRT-PCR) was performed on an ABI StepOnePlus PCR System (Applied Biosystems, Foster City, CA, USA). Data were normalized to α-Tubulin mRNA levels and were analyzed using the 2−ΔΔCt method. All primers used for qRT-PCR were purchased from Qiagen (Hilden, Germany). The primer sequences of qRT-PCR were as follows. ISOC1: 5′-AGCGTTAGCCGAGATTCCTG-3′, 5′- CCAGGGCAGTTTGCTGGATA-3′; Interleukin-1beta (IL-1Β): 5′-AATGCCACCTTTTGACAGTGA-3′, 5′-TCAGGACAGCCCAGGTCAA-3′; Interferon-gamma (IFN-γ): 5-AGCAAGGCGAAAAAGGATGC-3′, 5′- TCATTGAATGCTTGGCGCTG -3′; Interleukin-6 (IL-6): 5′-AGCCAGAGTCCTTCAGAGAGA-3′, 5′-GCCACTCCTTCTGTGACTCC-3′; Interleukin-12 (IL-12): 5′-CCAGCATGTGTCAATCACGC-3′, 5′-GAGACTGGAATGACCCTGGC-3′; PEX11B: 5′-TTCAGTGCTCAGAGCCAAGC-3′, 5′-CTAGCCCCATGTCTCTGCAA-3′; Peroxisomal Biogenesis Factor 3 (PEX3): 5′-AATTCCCATAGTGAACGGGCA-3′,

5′-AAGAACCACAGGTAAGCTGC-3′; PEX7: 5′-TAGTGGTGATGGCTCACTGC-3′, 5′-GTTGGATCCCACTCCTGCGT-3′; IL-4: 5′-CAACCCCCAGCTAGTTGTCA-3′; 5′-TGTCGCATCCGTGGATATGG-3′; IL-10: 5′-TGGACTCCAGGACCTAGACA-3′; 5′-CGGAGAGAGGTACAAACGAGG-3′; α-Tubulin: 5′-GCCCAACCTACACTAACCTAAACA-3′, 5′-CAACATTCAGGGCCCCATCAAA-3′.

### 3.4. Immunoblot and Immunoprecipitation

Cells were lysed with RIPA buffer (Beyotime, Shanghai, China). Protein samples were subjected to SDS-PAGE and transferred to PVDF membranes (Millipore, Bedford, MA, USA). The primary antibodies used included 1/1000 anti-α-Tubulin (AF0001), 1/1000 anti-pan Phospho-Serine/Threonine (anti-Pan-P) (AF5725), 1/500 anti-HA (AF5057) (Beyotime), 1/1000 anti-Isoc1 (PA5-67096), anti-PEX11B (PA5-37011), 1/500 anti-PEX1 (PA5-100677), 1/500 anti-PEX3 (PA5-42814), 1/500 anti-PEX7 (PA5-101744) (ThermoFisher Scientific, San Jose, CA, USA), 1/1000 anti-COX-2 (12282), 1/1000 anti-AKT (4691), 1/1000 anti-phospho-AKT (4060), 1/500 anti-IκBα (4812), 1/500 anti-phospho-IκBα (9246), 1/500 anti-extracellular signal-regulated kinase (ERK1/2) (4696), 1/200 anti-phospho-ERK1/2 (4370)(Cell Signaling Technology, Inc., Danvers, MA, USA), 1/500 anti-ATP-binding cassette sub-family D member 3 (ABCD3) (sc-514728) (ABCD3), 1/500 anti-PEX11B (PA5-37011), 1/500 anti-PEX1 (PA5-100677), 1/500 anti-PEX3 (PA5-42814), 1/500 anti-PEX7 (PA5-101744), and 1/500 anti-Ubiquitin (sc-271289) (Santa Cruz Biotechnology, Santa Cruz, CA, USA). Secondary Abs were purchased from Beyotime. Immunoblots were revealed using the SuperSignal west pico substrate (ThermoFisher Scientific, San Jose, CA, USA). A coimmunoprecipitation (Co-IP) experiment was modified from a previous protocol [35]. Briefly, cells were harvested and treated with lysis buffer (1.19% HEPES, 0.88% NaCl, 0.04% EDTA, 1% NP-40 (Beyotime Biotechnology, P0013F)) containing a protease inhibitor. Lysates were incubated overnight with the precipitating antibody anti-HA (Santa Cruz Biotechnology) diluted 1:200, followed by a 2-h incubation with 30 mL of protein A/G plus agarose affinity gel slurry. Immune complexes were then washed five times with lysis buffer, and then an immunoblotting analysis was conducted.

### 3.5. Immunofluorescence Assay

Cells were centrifuged at 100× *g* for 5 min, washed twice in cold PBS, and fixed in 4% paraformaldehyde for 15 min. Afterwards, cells were permeabilized with 0.5% Triton X-100 for 10 min and then blocked with Immunol Staining Blocking Buffer (Beyotime) for 2 h at room temperature. Subsequently, cells were washed three times with PBS for 5 min and incubated with primary antibodies overnight at 4 °C. Following incubation, cells were washed three times with PBS and incubated with secondary antibodies for 2 h at room temperature. Nuclei were stained with DAPI. Primary antibodies used in immunofluorescence assay include anti-ABCD3 and anti-ISOC1. Cells were observed with a fluorescence microscope, and fluorescence intensity was measured with ImageJ.

### 3.6. O2-Scavenging Capacity

O2- scavenging capacity was detected using a CheKine™ Superoxide anion Scavenging Capacity Assay Kit (Abbkine, Redlands, CA, USA) according to the manufacturer’s instructions.

### 3.7. Measurement and Quantification of Oxidative DNA Damage

An 8-hydroxy-2 deoxyguanosine (8-OHDG) competitive ELISA was performed using a commercial kit (OxiSelect Oxidative DNA Damage ELISA Kit (8-OHDG Quantitation)) (Cell Biolabs, San Diego, CA, USA) according to the manufacturer’s instructions.

### 3.8. Cell Proliferation Assay

Cell proliferation was assayed by CCK-8 assay (Beyotime) according to the manufacturer’s protocol. Briefly, cells were combined with 10 μL reagent of CCK-8 solution for 2 h incubation at 37 °C. The optical density was measured with a microplate reader (Bio-Rad, Hercules, CA, USA) at 450 nm.

### 3.9. Statistical Analysis

All data were from three biological replicates and expressed as the mean ± s.e.m. The statistical significance between groups was determined by two-tailed Student’s *t*-test or ANOVA, followed by Bonferroni post hoc test. A value of *p* < 0.05 was considered significant. GraphPad Prism software 8.0 was used for all data analysis.

## 4. Discussion

Inflammation is involved in the development of many diseases [36,37]. In the complex network of inflammation, there is little information about the role of ISOC1 in inflammation regulation [38]. In this study, we constructed a LPS-stimulated macrophage model to investigate the role of ISOC1 in natural immunity. We found that ISOC1 can regulate the inflammatory response by regulating AKT1/PEX11B and peroxisomes.

Current studies on ISOC1 mainly focus on cancers. For example, it has been reported that knockout of ISOC1 activates the AKT1/GSK-3β pathway and induces the apoptosis of colon cancer cells [38]. In our study, the inflammatory role of the ISOC1/AKT1 pathway was identified, and a new downstream factor regulated by AKT1—Pex11b—was proposed. PEX11B has been reported to mediate growth and division of mammalian peroxisomes [39]. PEX11B has been also shown to inhibit ZIKV replication, likely by increasing peroxisome numbers and enhancing downstream IFN-dependent antiviral signaling [38]. The depletion of M. tuberculosis Rv3034c decreases the expression of peroxins, such as PEX3, PEX5, PEX9, and PEX11B [40]. In addition, we showed that ISOC1 regulated peroxisomes, and this regulation is AKT1-dependent. In recent years, peroxisomes have become signaling hubs for inflammatory and antiviral defense pathways. Peroxisomes can produce and remove cellular active anions and participate in the metabolic process of inflammation-related substances, such as polyunsaturated fatty acid, prostaglandins, and leukotrienes [17]. Additionally, it has been proved that peroxisome may be an organelle necessary for the replication of ZIKV virus [24]. All of these factors indicate the role of peroxisome homeostasis in immune regulation. In this study, we found that the phosphorylation of AKT1 can regulate the stability of PEX11B through protein modification. We also identified the AKT1/PEX11B/peroxisome pathway, a new branch of AKT1 regulating the inflammatory response.

Some additional data could also be derived from this study. For example, after checking the two-way ANOVA analysis in Figure 4C–H, we found that AKT1 inhibition with Per, rather than NF-κB inhibition with BAY, reduced oxidative stress both in wild-type cells and Isoc1^-/-^ cells upon LPS stimulation. Interestingly, this result is also consistent with previous reports. For example, it has been reported that AKT1 inhibition reduced oxidative stress in cardiomyopathy rat models and colon cancer cells [41,42]; meanwhile, we found in another report that LPS regulated NF-κB through a ROS-independent mechanism in J774.1 cells [43]. In this study, we tested the expressions of M1 markers IL-6, IL-12, IL-1β, and IFNγ [44], and M2 markers IL-4 and IL-10 [45] in WT and Isoc1^-/-^ macrophages. From the results, we observed that after LPS infection, ISOC1 deletion increased expressions of M1 markers (Figure 1D), while it reduced expressions of M2 markers (Figure S1A). Therefore, we speculate that Isoc1 knockout is beneficial to M1 after LPS stimulation. Rare studies have reported the function of ISOC1 on macrophage’s polarity; however, a previous study from Gao et al. [12] found that ISOC1 knockout promotes p-STAT1 translocation to the nucleus in colon cancer cells. Since STAT1 is involved in M1 polarization [46], this indicates that ISOC1 knockout probably plays a role in M1 polarization, which is consistent with our observation that ISOC1 deletion promotes M1 polarization. 

However, several questions remain to be addressed: first, according a previous study, ISOC1 has both a predicted PTS1 and PTS2 sequences to target it to the peroxisomal matrix [9]. Does ISOC1 co-localize to peroxisomes? We thus performed fluorescent staining to detect the co-localization of ISOC1 and peroxisomes. Fluorescent staining results in Figure S2E showed that after LPS stimulation in WT cells, only a small part of ISOC1 can interact with peroxisomes, indicating the possibility that ISOC1 is free in the cytoplasm and may bind to AKT1 for its activation. However, this hypothesis needs to be confirmed with further experiments. Second, how does AKT1 regulate the protein modification of PEX11B? Since AKT1 is a serine/threonine protein kinase, we speculate that AKT1 may directly or indirectly affect the phosphorylation level of PEX11B, leading to the increase in its ubiquitination and degradation. Using several websites, we tried to predict direct phosphorylation sites in PEX11B with AKT1 via several websites. However, no predicted sites were identified. We thus suspect that the effect of AKT1 on PEX11B phosphorylation might be indirect, and this will need further experiments for verification. Last, PEX11B is important for peroxisome division [47]. However, the mechanism by which PEX11B phosphorylation modulates its ubiquitination remains unclear. It may be that after phosphorylation of PEX11B, the conformation of the protein changes, and the ubiquitination site is thus exposed. However, these hypotheses require further studies.

## Data Availability

Data sharing is not applicable to this article as no datasets were generated or analyzed during the current study.

## Supplementary Materials

The following supporting information can be downloaded at: https://www.mdpi.com/article/10.3390/molecules27185896/s1, Figure S1: ISOC1^-/-^ results a higher apoptosis rate and Isoc1 overexpression promotes inflammation after LPS treatment; Figure S2. Pex11b knockdown resulted in decreased peroxisomes and enhanced inflammatory response.
