# Supplementary material for: ISOC1 Modulates Inflammatory Responses in Macrophages through the AKT1/PEX11B/Peroxisome Pathway
Source: Molecules. 2022 Sep 11;27(18):5896. doi: 10.3390/molecules27185896 (PMC9505204; doi:10.3390/molecules27185896)
Supplement: Supplementary file 1 [file molecules-27-05896-s001.zip › molecules-1869981-supplementary.pdf]

Supplementary Materials for

ISOC1 modulates inflammatory responses in macrophages through the AKT1 /PEX11B/ peroxisome pathway

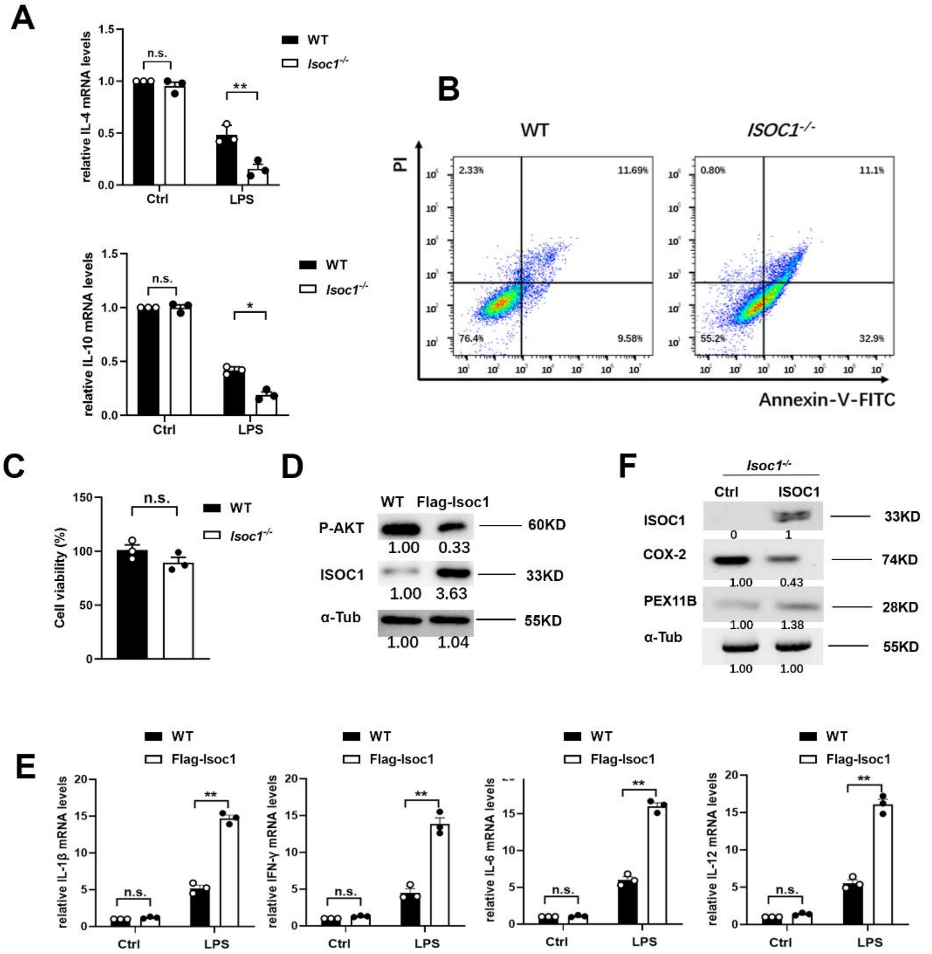

Figure S1. ISOC1$^{-/-}$ results a higher apoptosis rate and Isoc1 overexpression promotes inflammation after LPS treatment.

A, B Wild type and Isoc1 deficient RAW264.7 cells were stimulated with 300 ng/mL LPS for 24 hours. The mRNA levels of anti-inflammatory factors were detected by qRT-PCR (**A**), and the levels of apoptosis were detected by flow cytometry (**B**). **C** Cell proliferation was assayed by a CCK-8 kit in WT or *Isoc1*$^{-/-}$ cells after LPS treatment. **D, E** RAW264.7 macrophages were transfected with the Flag- labeled

Isoc1 vector for 12 hours, and were stimulated with 300 ng/mL LPS for 24 hours. The levels of ISOC1 and P-AKT1 were detected by WB (**D**), and mRNA levels of inflammatory factors was detected by qRT-PCR (**E**). **F** ISOC1 was overexpressed in *Isoc1*$^{-/-}$ cells after LPS treatment, and the levels of COX-2 and PEX11B were detected by WB. A single data point represented one technical repeat. All data are shown as mean ± s.e.m (n = 3). **P*<0.05, ***P*<0.01. Student t test or Two-way ANOVA followed by Bonferroni post *hoc* test was used for data analysis.

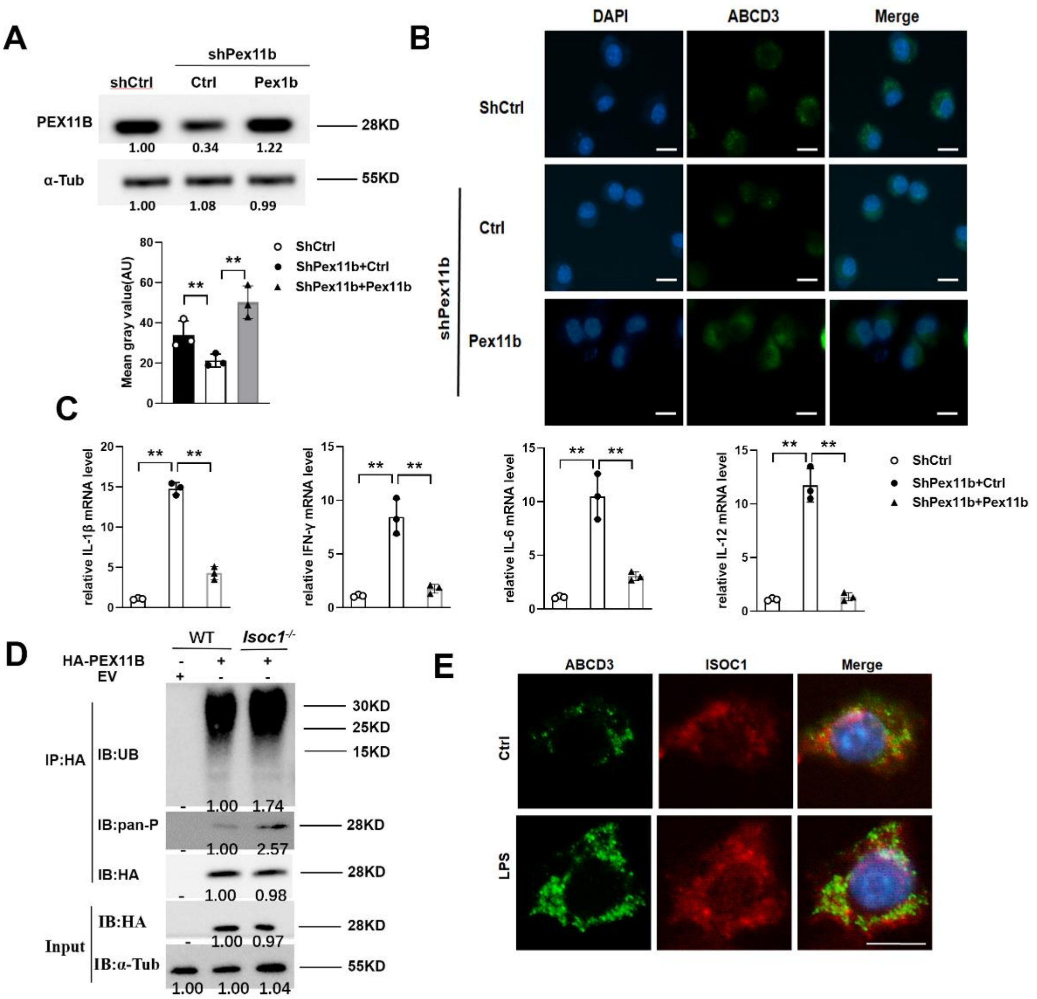

Figure S2. **Pex11b knockdown resulted in decreased peroxisomes and enhanced inflammatory response.**

A Knockdown efficiency of Pex11b was validated by WB. **B** Peroxisomes were labeled with ABCD3 and were observed with fluorescence microscopy after Pex11b knockdown upon LPS treatments. Scale bar = 10 μm. Mean gray value were analyzed by Image J. **C** qRT-PCR was used to detect the expression levels of IL-1β, IFN-γ, IL-6, and IL-12 after Pex11b knockdown upon LPS treatments. All data are shown as mean ± s.e.m. (n = 3). **D** WT or *Isoc1*$^{-/-}$ cells were transfected with pCMV-HA or HA-labeled PEX11B, and stimulated with LPS for 24h. Phosphorylation and ubiquitination levels of pex11b were detected by Co-IP. **E** Co-localization of peroxisomes (labeled with ABCD3) and ISOC1 in RAW 264.7 cells upon LPS treatment were observed with fluorescence microscopy. Scale bar =10 μm. $^{**}P<0.01$. Student t test was used for data analysis.
